# Afferent activity to necklace glomeruli is dependent on external stimuli

**DOI:** 10.1186/1756-0500-2-31

**Published:** 2009-03-02

**Authors:** Renee E Cockerham, Frank L Margolis, Steven D Munger

**Affiliations:** 1 Department of Anatomy and Neurobiology, University of Maryland School of Medicine, Baltimore, MD, USA

## Abstract

**Background:**

The main olfactory epithelium (MOE) is a complex organ containing several functionally distinct subpopulations of sensory neurons. One such subpopulation is distinguished by its expression of the guanylyl cyclase GC-D. The axons of GC-D-expressing (GC-D+) neurons innervate 9–15 "necklace" glomeruli encircling the caudal main olfactory bulb (MOB). Chemosensory stimuli for GC-D+ neurons include two natriuretic peptides, uroguanylin and guanylin, and CO_2_. However, the biologically-relevant source of these chemostimuli is unclear: uroguanylin is both excreted in urine, a rich source of olfactory stimuli for rodents, and expressed in human nasal epithelium; CO_2 _is present in both inspired and expired air.

**Findings:**

To determine whether the principal source of chemostimuli for GC-D+ neurons is external or internal to the nose, we assessed the consequences of removing external chemostimuli for afferent activity to the necklace glomeruli. To do so, we performed unilateral naris occlusions in *Gucy2d-Mapt-lacZ *^+/- ^mice [which express a β-galactosidase (β-gal) reporter specifically in GC-D+ neurons] followed by immunohistochemistry for β-gal and a glomerular marker of afferent activity, tyrosine hydroxylase (TH). We observed a dramatic decrease in TH immunostaining, consistent with reduced or absent afferent activity, in both necklace and non-necklace glomeruli ipsilateral to the occluded naris.

**Conclusion:**

Like other MOB glomeruli, necklace glomeruli exhibit a large decrease in afferent activity upon removal of external stimuli. Thus, we conclude that activity in GC-D+ neurons, which specifically innervate necklace glomeruli, is not dependent on internal stimuli. Instead, GC-D+ neurons, like other OSNs in the MOE, primarily sense the external world.

## Findings and discussion

The mammalian olfactory system responds to a multitude of chemical stimuli, including general odors that convey information about food, prey and predators, and semiochemicals such as pheromones and social cues that can influence stereotypical behaviors and hormonal states [1,2]. To accomplish this massive task, mammals employ a number of distinct olfactory subsystems, each of which may be tuned to particular subsets of olfactory stimuli [1]. For example, subpopulations of sensory neurons in the vomeronasal organ that express V2R-type receptors are responsive to MHC peptides [3], peptides found in glandular secretions [4], or lipocalin proteins present in urine (i.e., major urinary proteins or MUPs) [5]. Sensory neurons in the main olfactory epithelium (MOE) that respond to the natriuretic peptides uroguanylin and guanylin [6] and to the gas CO_2 _[7] can be distinguished from other cells by their specific expression of the guanylyl cyclase GC-D, the phosphodiesterase PDE2, and the cyclic nucleotide-gated channel subunit CNGA3 [1,8-10].

Olfactory recognition of semiochemicals that signal between conspecifics may present a particular challenge, since these compounds can be produced internally, including within the nasal cavity of the recipient animal. For example, some MUPs (MUP4 and MUP5) are expressed in mouse nasal glands [11]. The stimuli that activate GC-D-expressing (GC-D+) neurons can have both exogenous and endogenous sources: CO_2 _is found in both inspired and expired air, while uroguanylin, which is secreted in urine, is expressed in mammalian intestine and kidney [12] and in human nasal mucosa [13]. The multiple sources of stimuli for GC-D+ neurons raise the questions of whether activity in these cells depends on external or internal stimuli, and whether these cells function as olfactory sensory neurons or as internal chemosensors.

**Figure 1:**
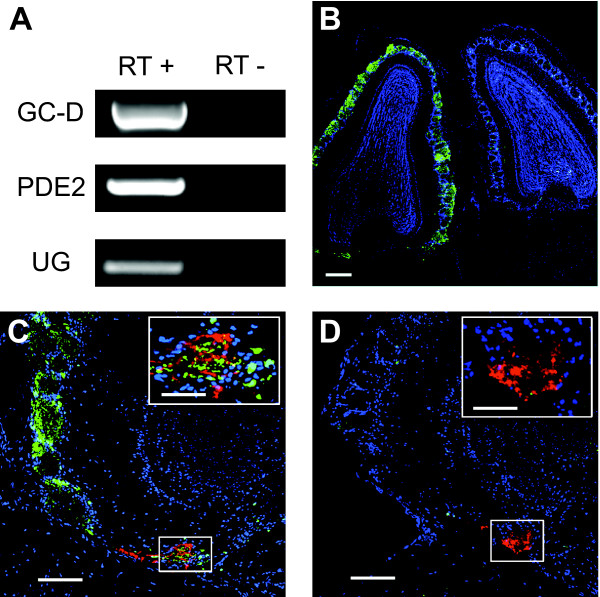
**Afferent activity to necklace glomeruli is dependent on exogenous stimulation of GC-D+ neurons**. **(A) **RT-PCR of GC-D, PDE2 and uroguanylin from nasal epithelium cDNA of C57BL/6J mice with or without reverse transcriptase (RT). The identity of each product was confirmed by sequencing. **(B) **Immunohistochemical staining of horizontal sections of left (non-occluded side) and right (occluded side) MOBs of a *Gucy2d-Mapt-lacZ *^+/- ^mouse. TH, green; DAPI, blue. Scale bar: 100 μM. **(C) **Immunohistochemical staining of left (non-occluded side) MOB of a naris occluded *Gucy2d-Mapt-lacZ *^+/- ^mouse. *Inset*, higher magnification view of area represented in box. β-gal, red; TH, green; DAPI, blue. Scale bars 50 μM, 20 μM (inset). **(D) **Right (occluded side) MOB of same mouse as in (C). *Inset*, higher magnification view of area represented in box. β-gal, red; TH, green; DAPI, blue. Scale bars 50 μM, 20 μM (inset).

We first asked if uroguanylin and/or guanylin are expressed in the nasal cavity of mice. We used reverse transcription-polymerase chain reaction (RT-PCR) to amplify uroguanylin and the GC-D+ neuron markers GC-D and PDE2, from cDNA reverse transcribed from total RNA extracted from mouse nasal mucosa (Figure 1A). No products were amplified from samples that were not reverse transcribed. We were unable to amplify a guanylin product with any of four different primer pairs. These results suggest that, as in humans [13], uroguanylin is expressed in the nasal cavity of the mouse.

GC-D+ neurons are activated by urine [6], which contains uroguanylin [12]. Nonvolatile stimuli present in urine, such as peptides, can access the MOE during normal social investigations [14], indicating that urine-derived uroguanylin could be available for stimulation of GC-D+ neurons. However, the presence of uroguanylin message in the MOE suggests that GC-D+ neurons could also be activated *in vivo *by endogenous uroguanylin. Similarly, the GC-D+ neuron stimulus CO_2 _is present in both inspired and expired air. To determine if GC-D+ neuron activity is primarily due to stimulation by compounds that originate from the external environment, we performed unilateral, non-reversible naris occlusions in adult *Gucy2d-Mapt-lacZ*^+/- ^mice. The *Gucy2d-Mapt-lacZ *line has a large section of the *Gucy2d *gene (which encodes GC-D) replaced by a construct expressing a tau-β-galactosidase (β-gal) reporter through gene-targeting [6]. Thus, GC-D+ neurons in heterozygous *Gucy2d-Mapt-lacZ *mice express GC-D from the wildtype allele, the β-gal reporter from the targeted allele, and exhibit wildtype chemoresponses [6]. Unilateral naris occlusion eliminates orthonasal stimulation of the ipsilateral MOE by external stimuli [15,16]. Retronasal stimulation via the nasopharynx, such as by components of expired air, is at least partially maintained with unilateral naris occlusion (e.g., [17,18]), though alteration of normal retronasal airflow patterns seems likely. The presence of GC-D+ neurons in several regions of the MOE, including the dorsal recesses, endoturbinates and septum [6-8,19], strongly suggests that GC-D+ neurons on the occluded side would still have access to retronasal stimulation.

To assay afferent activity to the necklace glomeruli, we took advantage of the correlation between afferent activity to MOB glomeruli and tyrosine hydroxylase (TH) expression levels in periglomerular interneurons. Disruption of MOE stimulation by naris occlusion or disruption of sensory neuron function by chemical ablation or gene deletion results in a large decrease in TH immunoreactivity surrounding MOB glomeruli (e.g., [6,20-24]). For example, TH immunoreactivity in necklace glomeruli is specifically reduced in *Gucy2d-Mapt-lacZ *^-/- ^mice, which lack a functional GC-D protein [6]. We have observed that necklace glomeruli exhibit extensive intrabulbar connections with canonical glomeruli through juxtaglomerular interneurons [25]. However, disruption of afferent activity to canonical glomeruli does not reduce TH immunoreactivity in necklace glomeruli [20]. Therefore, TH levels in necklace glomeruli-associated periglomerular cells are correlated with direct sensory input of GC-D+ neurons to the necklace glomeruli.

Fourteen days after naris occlusion we assessed TH expression in canonical and necklace glomeruli by double label immunohistochemistry for TH and β-gal (n = 5 mice). TH levels remained high on the non-occluded side (Figure 1B, C), but were dramatically reduced in both canonical and necklace glomeruli on the occluded side (Figure 1B, D). TH immunoreactivity was considerably reduced in all β-gal-immunopositive (i.e., necklace) glomeruli (data not shown). These results indicate that internal GC-D+ neuron stimuli are insufficient to maintain normal afferent activity in these cells and that GC-D+ neurons are olfactory sensory neurons primarily activated by stimuli that enter the nose from the external environment.

## Methods

### Mice

The generation and characterization of *Gucy2d-tau-lacZ *^+/- ^mice was described elsewhere [6] (the *Mapt-lacZ *reporter construct expresses a tau-β-gal fusion protein under control of the *Gucy2d *reporter). Mice were individually housed in plastic cages (28 × 17.5 × 13 cm) with stainless steel wire lids. Food and water was available *ad libitum*. Mice used in experiments were 30–100 days old. All animal procedures were approved by the Institutional Animal Care and Use Committee of the University of Maryland School of Medicine.

### RT-PCR

MOE tissue was dissected from C57BL/6J mice (n = 5), pooled, and total RNA was extracted with Trizol reagent (Invitrogen). cDNA was reverse-transcribed from 2 μg of total RNA using oligo(dT) primers, and the resulting cDNA was used as template for PCR amplification with TaqPro Complete (Denville Scientific, Metuchen, NJ). Primers were designed using MacVector software. All PCR products (amplicons) were purified, subcloned into sequencing vectors, and sequenced by the University of Maryland School of Medicine Biopolymer Lab.

### Naris occlusion

*Gucy2d-Mapt-lacZ *^+/- ^adult male mice were anesthetized by intraperitoneal injection of Nembutal (50mg/kg body weight). The right naris of each mouse was occluded by electrocautery [26,27]; the left, non-occluded side served as an internal control. A layer of Super Glue (Super Glue Corp, Rancho Cucamonga, CA) was administered over the closed naris to ensure full closure and to prevent reopening. After cauterization, the animals were kept warm and monitored until recovery, and daily thereafter. Efficacy of the occlusion was evaluated by tyrosine hydroxylase (TH) immunohistochemistry in multiple canonical glomeruli in the MOB, and mice in which the occlusion was incomplete (as indicated by equivalent TH staining on the occluded and non-occluded sides or by visual inspection of the naris) were not analyzed further.

### Immunohistochemistry

Double label immunohistochemistry for β-gal and TH was performed as previously published [6]. At least 14 days after naris occlusion, mice (n = 5) were intracardially perfused with 0.9% saline followed by 4% paraformaldehyde in 0.1 M sodium phosphate buffer (pH 7.4). Brains, including MOBs, were removed and post-fixed for 2 hours at 4°C in the same fixative. Tissue was cryoprotected in 10% sucrose/0.1 M PBS for 2 hours, 20% sucrose/0.1 M PBS for 2 hours, and 30% sucrose/0.1 M PBS overnight (all at 4°C). Paired MOBs were sectioned by cryostat in the horizontal plane at 16 μm, and the sections collected onto glass slides. Sections were blocked with 2% heat inactivated donkey serum and 0.2% Triton X-100 in 0.1 M PBS (pH 7.4) (blocking solution) at room temperature for 2 hours. Sections were then incubated overnight at room temperature with rabbit anti-tyrosine hydroxylase (1:500; Chemicon) and mouse anti-β-gal (1:2000; Chemicon) in blocking solution.

Immunoreactivity was visualized with appropriate Cy2-conjugated anti-rabbit and Cy3-conjugated anti-mouse secondary antibodies. Sections were visualized with an Olympus Fluoview 500 confocal microscope. Confocal images were processed using Olympus Fluoview v5.0 software.

## Competing interests

The authors declare that they have no competing interests.

## Authors' contributions

REC and SDM conceived of the study, designed the experiments, analyzed the results and read and approved the final manuscript. FLM performed the naris occlusions and assisted with the experimental analysis. REC performed the experiments and wrote the paper. SDM and FLM edited the paper. All authors read and approved the final manuscript.
